# Systemic Sirolimus Monitoring in Pediatric Pulmonary Vein Stenosis

**DOI:** 10.1007/s00246-025-04038-6

**Published:** 2025-10-01

**Authors:** Heather Meluskey, Bridget Blowey, Anna B. O’Brien, Michael L. O’Byrne, Jonathan J. Rome, David B. Frank, Catherine M. Avitabile, Mudit Gupta, Jessica Tang, Kimberly L. Butler, Constantine D. Mavroudis, Stephanie Fuller, Ryan Callahan

**Affiliations:** 1 Division of Cardiology, The Children’s Hospital of Philadelphia and Department of Pediatrics, Perelman School of Medicine at the University of Pennsylvania, Philadelphia, PA USA; 2 Department of Pharmacy, The Children’s Hospital of Philadelphia, Philadelphia, PA USA; 3 Clinical Futures, The Children’s Hospital of Philadelphia, Philadelphia, PA USA; 4 Department of Cardiovascular and Thoracic Surgery, Dell School of Medicine at the University of Texas, Austin, TX USA; 5 Division of Cardiothoracic Surgery, The Children’s Hospital of Philadelphia and Department of Surgery, Perelman School of Medicine at the University of Pennsylvania, Philadelphia, PA USA; 6 The Children’s Hospital of Philadelphia, 3401 Civic Center Blvd, Philadelphia, PA 19104 USA

**Keywords:** Congenital heart disease, Medical therapy, In stent restenosis, mTOR inhibition

## Abstract

Systemic sirolimus (SS) is an mTOR inhibitor used in the management of pediatric intraluminal pulmonary vein stenosis (PVS). SS initiation, monitoring, including patient compliance with toxicity surveillance, and potential adverse events (AE) in PVS patients are under reported. A single-center retrospective cohort study of consecutive patients who were initiated on SS for PVS from January 1, 2020 to December 31, 2024 was performed. Fifty patients with a median age of 7 months (range 2–165) received SS for PVS (median number of stenotic veins; *n* = 3 (1–4)) for a median duration of 18 months (1–60). The median time to therapeutic level was 9 days [IQR 3, 20] with two never achieving therapeutic values. In patients who received SS for at least 6 months (*n* = 39), the median number of blood draws and number of dose adjustments in the first 6 months were 14 [IQR 5, 27] and 3 [1, 7], respectively. Most levels among patients (75%; [IQR 64, 84]) did not require a dose adjustment. Toxicity surveillance compliance increased from 58% [IQR 42, 83] to 79% [IQR 62.5, 92] (*p* = 0.22) following transitioning ownership of SS management to a dedicated PVS team. Eighteen percent (9/50) of patients had an AE potentially related to SS; SS was discontinued in three. PVS patients receiving SS have high, but variable rates of therapeutic levels and SS discontinuation due to AEs is uncommon. Compliance with safety labs may improve with ownership by a dedicated monitoring team.

## Introduction

Intraluminal pediatric pulmonary vein stenosis (PVS) is an aggressive disease with historically poor outcomes. Efforts from individual centers implementing resource-intensive multidisciplinary interventional and medical therapy have dramatically improved medium-term outcomes [1–3]. One potentially important component of this regimen is systemic sirolimus (SS), a medication with anti-proliferative and immunosuppressive properties through inhibition of the mammalian target of Rapamycin (mTOR). SS has been used empirically in the hopes of mitigating in stent restenosis and to reduce the risk of upstream PVS progression with promising observational results [1, 3–5].

Previous work has demonstrated the feasibility of SS therapy in pediatric PVS patients, though studies to date have been limited to either short treatment durations (8 weeks) [4] or small sample sizes focused primarily on patient outcomes (*n* = 15^1^, *n* = 10^5^). To our knowledge, no prior investigations have evaluated the initiation and maintenance of SS therapy in this population. This is particularly important because most eligible patients are complex and medically fragile infants, in whom the therapeutic window is narrow and optimal dosing is variable. Further, coordination of therapy requires substantial clinical oversight. Institutions with centralized PVS programs and sufficient patient volume may offer an opportunity to streamline care, consolidate expertise, and support primary cardiologists who may have variable comfort with this agent. Given these challenges, this study aims to describe our institutional experience with SS therapy in a high-volume PVS center, specifically evaluating time to therapeutic level, monitoring trends including time in therapeutic range (TTR) and patient compliance with toxicity surveillance, and the incidence of potential drug-related AEs.

## Materials and Methods

### Study Population

**Fig. 1:**
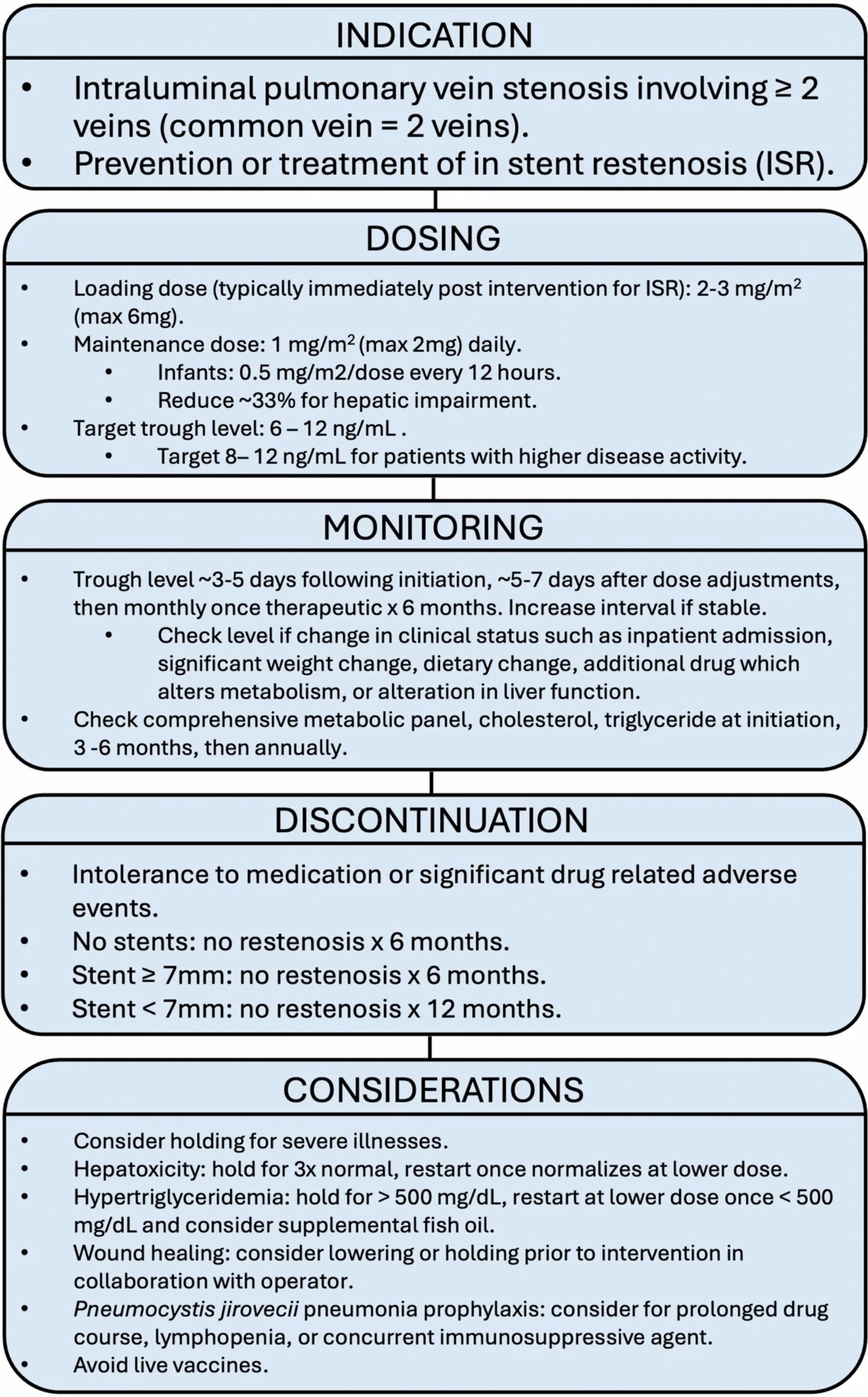
Systemic Sirolimus Therapy Institutional Protocol Overview

A single-center retrospective cohort study was performed. All consecutive patients who were initiated on SS for the indication of PVS and were followed by the Children’s Hospital of Philadelphia PVS team from January 1, 2020 to December 31, 2024 were eligible for the study. Figure 1 details the institutional SS therapy protocol. The study was determined to be exempt from review by our center’s institutional review board under the Common Rule. This retrospective chart review study involving human participants was in accordance with the ethical standards of the institutional and national research committee and with the 1964 Helsinki Declaration and its later amendments or comparable ethical standards.

### Study Measures

Data were abstracted from the institutional electronic medical record. Patient demographics as well as data on drug initiation, serial drug level monitoring, TTR, drug toxicity surveillance compliance, and AEs potentially related to SS were collected. AEs potentially related to SS included known drug side effects requiring collaborative discussion between the primary medical providers and the PVS team with consideration of SS dose modification or discontinuation. Adverse events were graded using the National Cancer Institute’s Common Terminology Criteria for Adverse Events Version 5.0 criteria [6]. For drug initiation, data included the duration from first dose to therapeutic level and the number of blood draws and dose adjustments needed to achieve a therapeutic trough level (6 to 12 ng/mL). For drug monitoring, the total number of blood draws and the number of drug levels that required a dose adjustment were collected. A common metric named time in the therapeutic range was modified from published data [7] and calculated as number of levels not requiring a dose change/total number of levels × 100%. Data on limus (sirolimus analogs including everolimus or zotarolimus) drug-eluting stent placement was collected to determine its potential influence on drug levels. Drug toxicity surveillance compliance reported the percent of expected laboratory tests during the first 6 months of therapy. This included 12 laboratory tests: monthly sirolimus levels as well as baseline and follow-up comprehensive metabolic panel, triglyceride levels, and cholesterol levels. The percent compliance was calculated as follows: (number of actual tests/12 expected tests) × 100%. Patients receiving SS for less than 6 months or those with incomplete data were excluded from this calculation. TTR and compliance pre- (era 1) and post- (era 2) transition of SS management to a dedicated PVS monitoring team (nurse practitioner, nurse coordinator, clinical pharmacist) in September 2023 was calculated. Prior to September 2023, the PVS team recommended SS initiation and followed the patients closely but left ownership of SS monitoring to the primary medical providers.

### Statistical Analysis

The cohort was described using conventional descriptive statistics; frequencies and percentages for categorical variables and medians, ranges, and interquartile ranges (IQR) for continuous variables. A Mann–Whitney *U* test was used to compare time to therapeutic level by loading dose use, and TTR and drug toxicity surveillance compliance by era. A *p*-value of < 0.05 was considered statistically significant.

## Results

### Study Population

Fifty patients with a median age of 7 months (range 2–165) were initiated on SS for the indication of PVS (median number of stenotic veins; *n* = 3 (1–4)) during the study period (Table 1). Most patients had congenital heart disease (30/50; 60%) including six with total anomalous pulmonary venous connection (12%).

**Table 1:** Patient Characteristics

Characteristics *n* = 50*	Number (%) or Median (Range)
Age at systemic sirolimus initiation (months)	7 (2, 165)
Body surface area (m^2^) (*n* = 47)	0.33 (0.18, 1.25)
Female	21 (42)
Cardiac diagnosis	
Structurally normal heart	17 (34)
Single ventricle	15 (30)
Left to right shunt	9 (18)
Total anomalous pulmonary venous connection	6 (12)
Other	3 (6)
Pulmonary vein stenosis diagnosis	
Congenital heart disease	30 (60)
Prematurity	16 (32)
Primary/Congenital**	3 (6)
Isolated lung disease	1 (2)
Comorbidities/history of:	
Prematurity (< 37-week gestation)	23 (46)
Lung disease***	25 (50)
Aspiration	14 (28)
Genetic syndrome	6 (12)
Number of stenotic veins	3 (1, 4)
Bilateral disease	37 (74)

*Unless otherwise specified

**Born full term, no cardiac defects or lung disease

***Bronchopulmonary dysplasia, chronic lung disease, airway abnormality, congenital diaphragmatic hernia

### Drug Initiation and Monitoring

**Fig. 2:**
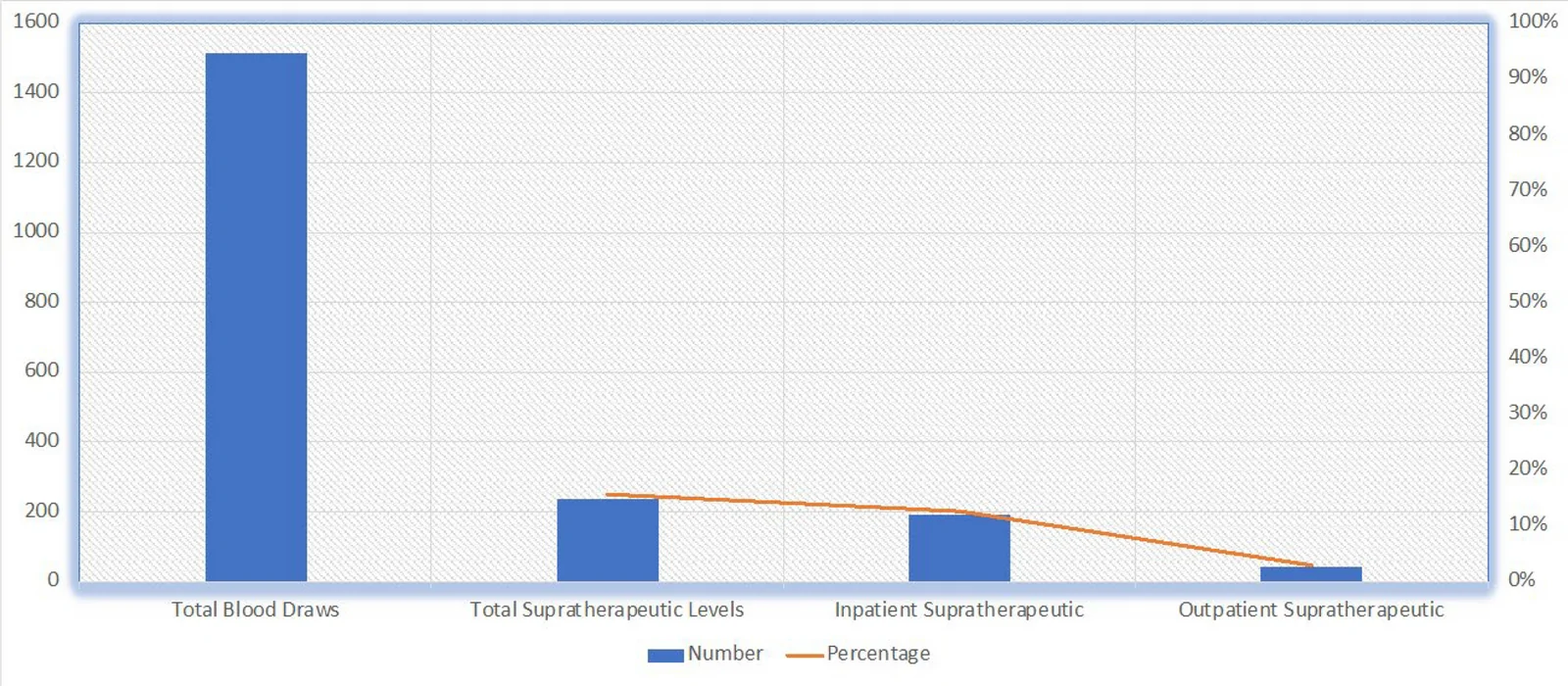
Bar graph demonstrating total number of sirolimus blood draws during study period as well as the number and percentage of supratherapeutic levels separated into location

Forty-four patients had complete data to determine the time to therapeutic level (Table 2). This occurred at a median of 9 days [IQR 3, 20] in 42 patients. Two patients never became therapeutic. A loading dose (2 mg/m^2^) was given in 13 patients (30%). There was no difference in the time to therapeutic level in patients who did (median 9.5 days [IQR 5, 38]) and did not (median 9 days [IQR 3, 15]) receive a loading dose (*p* = 0.23). Over the course of 1,101 patient months on SS, 1,513 levels were drawn with a median level of 7.8 ng/mL [IQR 5.8, 10.3]. Most levels (875/1513; 58%) were within goal range and rarely resulted in a dose adjustment (21/875; 2.4%). Of the levels either above or below goal range, 55% (351/638) resulted in a dose change. Most of the supratherapeutic levels occurred while inpatient (192/237; 81%) (Fig. 2). The median TTR for all patients was 75% [IQR 64, 84]. There was an increase in the median TTR from era 1 (69% [IQR 63, 86]) to era 2 (80% [IQR 69, 87] although this was not statistically significant (*p* = 0.15). Duration of therapy and discontinuation reasons are reviewed in Table 2.

**Table 2:** Sirolimus Characteristics

Characteristics *n* = 50*	Number (%) or Median (Range) or [IQR]
Loading dose given (*n* = 44)**	13 (30)
Time to therapeutic level (days) (*n* = 42)***	9 [3, 20]
# of dose adjustments before therapeutic	0 [0, 2]
# of blood draws before therapeutic	2 [1, 3]
All levels (*n* = 1513)	7.8 [5.8, 10.3]
Below goal range (< 6 ng/mL)	401 (26)
Dose change made	218 (54)
Above goal range (> 12 ng/mL)	237 (16)
Dose change made	133 (56)
Within goal range (6–12 ng/mL)	875 (58)
Dose change made	21 (2)
Duration of therapy (months)	18 (1, 60)
# of patient months on drug	1101
# of patients currently receiving drug	30 (60)
Drug discontinuation reason (*n* = 20)	
Disease stabilization	9 (45)
Patient death	6 (30)
Potential drug adverse event	3 (15)
Completed planned 8-week course	2 (10)

*Unless otherwise specified

**Six patients with incomplete data

***Two patients never became therapeutic

There were twenty instances where a limus drug-eluting stent was implanted, and the patient had a subsequent drug level within 7 days. Three levels were supratherapeutic (15%; 95% CI: 3.2–38%), 9 were subtherapeutic (45%; 95% CI: 23–68%), and 8 were therapeutic (40%; 95% CI: 19–64%). Two of the 3 supratherapeutic levels did not require a dose adjustment.

In patients with complete data who received SS for at least 6 months (*n* = 39), the median number of blood draws and number of dose adjustments in the first 6 months were 14 [IQR 5, 27] and 3 [IQR 1, 7], respectively (Table 3). More blood draws and dose adjustments occurred while inpatient than while outpatient. The median toxicity surveillance compliance increased from era 1 (58% [IQR 42, 83]) to era 2 (79% [IQR 63, 92]) although this was not statistically significant (*p* = 0.22).

**Table 3:** Sirolimus Characteristics for Six-Month Cohort

Characteristics *n* = 39	Number (%) or Median [IQR]
# of blood draws	14 [5, 27]
As inpatient	10 [1, 25]
As outpatient	1 [0, 3]
# of dose adjustments	3 [1, 7]
As inpatient	4 [1, 8]
As outpatient	1 [0, 1]
Toxicity surveillance compliance %*	67 [42, 92]
Before dedicated PVS team managing drug	58 [42, 83]
After dedicated PVS team managing drug	79 [62.5, 92]

Patients initiated sirolimus at The Children’s Hospital of Philadelphia and received sirolimus for minimum of six months. Data limited to first six months of therapy

*PVS * pulmonary vein stenosis

*Toxicity surveillance laboratory test expectation = baseline and follow-up triglyceride, cholesterol, comprehensive metabolic panel (6), and monthly sirolimus levels for 6 months (6). Compliance = (number of actual laboratory tests / number of expected laboratory tests) × 100%

Nine patients (18%) experienced an AE potentially related to SS therapy which included hypertriglyceridemia (*n* = 4; Grades 2,3,3,4), lung infection (*n* = 2; Grades 2,3), oral mucositis (*n* = 2; Grades 2,3), and delayed wound healing (*n* = 1; Grade 2). Only the patients with oral mucositis developed their AE in the setting of supratherapeutic levels. In the patients with hypertriglyceridemia, two discontinued SS and two successfully restarted SS at lower target levels with supplemental fish oil. One patient acquired *Pneumocystis jirovecii* pneumonia (PJP), developed pulmonary vein restenosis after discontinuing SS, and was subsequently restarted with concurrent PJP prophylaxis. One patient developed a lung abscess and has remained off SS. One patient with history of prematurity, severe bronchopulmonary dysplasia, adrenal insufficiency requiring stress dose steroids, and surgical necrotizing enterocolitis underwent exploratory laparotomy, lysis of adhesions, enterocutaneous fistula/ostomy closure, and cholecystectomy. SS was not held, and the preoperative level was 6.3 ng/mL. On postoperative day 6, the patient developed cellulitis with early evidence of wound dehiscence at the incision site. SS was held and the wound was managed conservatively with antibiotics. SS was restarted on postoperative day 14 once the wound adequately healed.

## Discussion

This single-center retrospective cohort study of children with PVS receiving SS demonstrated a reasonable time to reach therapeutic levels, with most levels not requiring dose adjustments over the course of therapy. Additionally, transitioning SS oversight to a dedicated PVS team corresponded with improvements in toxicity surveillance compliance and TTR, though differences between care eras did not reach statistical significance. Lastly, SS discontinuation because of AEs potentially related to SS was uncommon.

These findings provide practical guidance for the implementation of SS in a vulnerable, high-risk population. Clinicians initiating SS in infants with PVS may face uncertainty around dosing, monitoring frequency, and risk of adverse events. Our data help fill this gap by characterizing the timeline to therapeutic levels, frequency of dose adjustments, duration, and typical monitoring burden, particularly during the first 6 months of therapy. Importantly, a structured, multidisciplinary monitoring approach may improve compliance with safety labs and may help maintain patients in therapeutic range. However, there are key areas for institutional quality improvement given that TTR and compliance remain imperfect, and challenges such as communication with families, non-adherence to recommended testing, and difficulties in coordinating timed lab draws persist.

Multiple factors, including the timing of blood draws, can affect the trough level of SS. Young children have individual variability in drug metabolism and higher clearance rates of SS as compared to adults [8, 9]. Additionally, drug–drug interactions, particularly with CYP3A and p-glycoprotein inhibitors or inducers, and dietary factors can influence SS levels [8, 9]. These factors are considered during drug initiation, ideally under the consultation of a clinical pharmacist. The specific timing required for trough level measurement poses a significant challenge for both medical providers and families, necessitating education and logistical planning. While it was not feasible to determine the timing of all blood draws in this study, nearly half of subtherapeutic or supratherapeutic levels did not result in a dose adjustment, suggesting that many may have been inappropriately timed and not true trough levels. Inpatient settings accounted for more blood draws and dose adjustments than outpatient assessments, likely due to increased exposure to factors that influence SS levels. Acute illness, dietary changes, and new drug–drug interactions are more common in inpatients and require closer monitoring. Notably, most supratherapeutic levels in this cohort were observed in inpatient samples. An additional consideration is the placement of a limus drug-eluting stent, a type of catheter-based pulmonary vein intervention, which may lead to measurable SS levels, as seen in infants undergoing ductal stenting. [10, 11] Interestingly, in our cohort, among patients who had SS levels within seven days following stent placement, only one required a dose change for a supratherapeutic level.

SS was well tolerated overall with few serious AEs necessitating dose reduction or discontinuation. This is consistent with the previous publications of SS use in PVS and in other pathologies [1, 4, 5, 12]. For example, a prospective trial of SS for the treatment of vascular anomalies reported 23 AEs in 60 patients, most of which were myelosuppression (16) and only 4 patients required dose reduction or discontinuation [12]. It is noteworthy that one of our patients acquired PJP and is now on antibiotic prophylaxis. Despite this occurrence, and because PJP with mTOR inhibitors is rare (prevalence 0.4% to 0.9%), we discuss prophylaxis on a case-by-case basis with consideration of the duration of therapy, lymphocyte count, and concurrent treatment with additional immunosuppressive medications including systemic corticosteroids [13–15]. For serious AE prevention, SS may be temporarily lowered or held for significant infections to optimize the immune response or prior to surgery to optimize wound healing, although this is debated [16, 17]. In practice, preferences on holding SS and timing of reinitiation for surgical procedures are at the discretion of the operator. Typically, for open heart surgery, SS is held two weeks prior to surgery and initiated at least seven days after chest closure.

SS is one of the medical therapies in the management of PVS and is used in addition to interventions to treat stenosis, surveillance testing, and removal of possible PVS triggers [18]. At the time of PVS diagnosis, workup and treatment for aspiration or other lung insult was performed, and normalizing pulmonary blood flow is considered to minimize the flow disturbance in the pulmonary veins [19–22]. Following transcatheter or surgical relief of PVS, medical therapy is initiated.

## Limitations

The retrospective nature of the study likely limited the detection of less serious AEs related to SS such as transient gastrointestinal issues or subclinical marrow suppression. Further, causality of all clinical events and the potential attributability to SS cannot be determined without prospectively followed treatment and control groups. This study presents the clinically significant events warranting collaborative SS management decisions which were documented by the PVS team throughout the study period. Intentional pauses in SS therapy and subsequent non-therapeutic levels without dose adjustments were not accounted for in this study. Future work focusing on clinical effectiveness of SS could consider TTR as a predictor variable.

## Conclusion

Pediatric patients receiving SS for PVS can achieve therapeutic levels in a reasonable timeframe and have high rates of therapeutic levels throughout therapy, though this varies among individuals and inpatient status. AEs potentially related to SS are generally manageable, with few resulting in drug discontinuation. Structured multidisciplinary oversight may improve TTR and compliance with safety labs, though further optimization is needed to enhance adherence and minimize variability in care.

## Data Availability

The data that support the findings of this study are available from the corresponding author upon reasonable request.
